# Watching jellyfish

**DOI:** 10.7554/eLife.52669

**Published:** 2019-10-25

**Authors:** Eve Marder

**Affiliations:** 1 Volen CenterBrandeis UniversityWalthamUnited States; 2 Biology DepartmentBrandeis UniversityWalthamUnited States

**Keywords:** Living Science, jellyfish, water temperature

## Abstract

The natural world is more complex, and also more fragile, than it appears.

Eleven years ago, my husband and I moved from the middle of the Back Bay in Boston to a condominium on the Waterfront, just outside of the North End. The North End is one of the last of the old neighborhoods in Boston, with restaurants, bakeries, home-made pasta makers, coffee shops, boutiques, a real butcher shop, interspersed with the now ubiquitous nail salons and banks. So, we are still 'in the city'. That said, our apartment is on a wharf that extends into the Boston Harbor, and our balcony and windows bring the harbor into our lives. We overlook the Boston Coast Guard station, and my husband has become an expert on the vessels that come and go. We watch the ice breakers, the buoy tenders, and the larger ships that are used to patrol the waters from Maine to the Caribbean, and we get to see training exercises in which new recruits learn how to maneuver smaller boats and perform water rescues.

As fascinating as the exploits of the Coast Guard are, it is the waters of the harbor that are the most mysterious. The first year we were here the harbor was filled with what seemed to be millions of jellyfish by late spring/early summer. They appeared one day, they got larger for a few weeks and then, just as suddenly, disappeared. The common or moon jellyfish, *Aurelia aurita*, is very beautiful and quite ethereal as it drifts in the harbor, moving with the waves and tides.

Now each spring, I look for the jellyfish, as some years there are few, some years apparently none, and every so often, a major visitation. If I were a real biologist or environmentalist, as are some of you, I would have known immediately whether seeing large numbers of jellyfish in the Boston Harbor is something I should welcome or worry about. Actually, jellyfish supposedly live permanently in the harbor, and larger blooms occur when the water temperature and salinity and over-fishing support their presence. This occurs because some fish prey on young jellyfish, and large numbers of jellyfish can result from the decreased presence of their normal predators and warmer temperatures. Moreover, jellyfish can survive at low oxygen levels, which favors their survival in sometimes polluted warmer waters. Thus, the apparent absence of jellyfish may signal the relative health of the harbor, or a measure of the water temperature of the preceding weeks. Large blooms of jellyfish can also have deleterious effects on the harbor as they prey on a variety of other species, which can contribute to a loss of fish stocks.

While there were few jellyfish this year, in the heat of the summer, I looked down at the harbor and saw dead carp floating belly-up in the water. There was an environmental crisis in the Charles River, which feeds directly into the harbor near us. Carp are particularly sensitive to heat and hypoxia, so the presumption is that the heat and low water levels resulted in these deaths. I have become preoccupied with the health of the harbor, both as I sit on my balcony or look out my window, and because my lab has been studying the effects of temperature on the nervous system of crabs, which also come from the Atlantic waters around Boston.

For many years we purchased crabs for experiments from fishermen (through a sea-food distributor) and kept them in controlled temperature sea-water tanks and studied them under controlled temperature conditions. About 10 years ago we became interested in studying animal-to-animal differences in resilience, and started to explore how the animals responded to changes in the water temperature. We study the effects of the temperatures that the animals normally experience, as well as the effects of temperatures warmer than those they have been exposed to in the past. Consequently, we now see in the laboratory the long-term effects of the yearly temperature fluctuations of the waters off the coast of New England.

**Figure 1:**
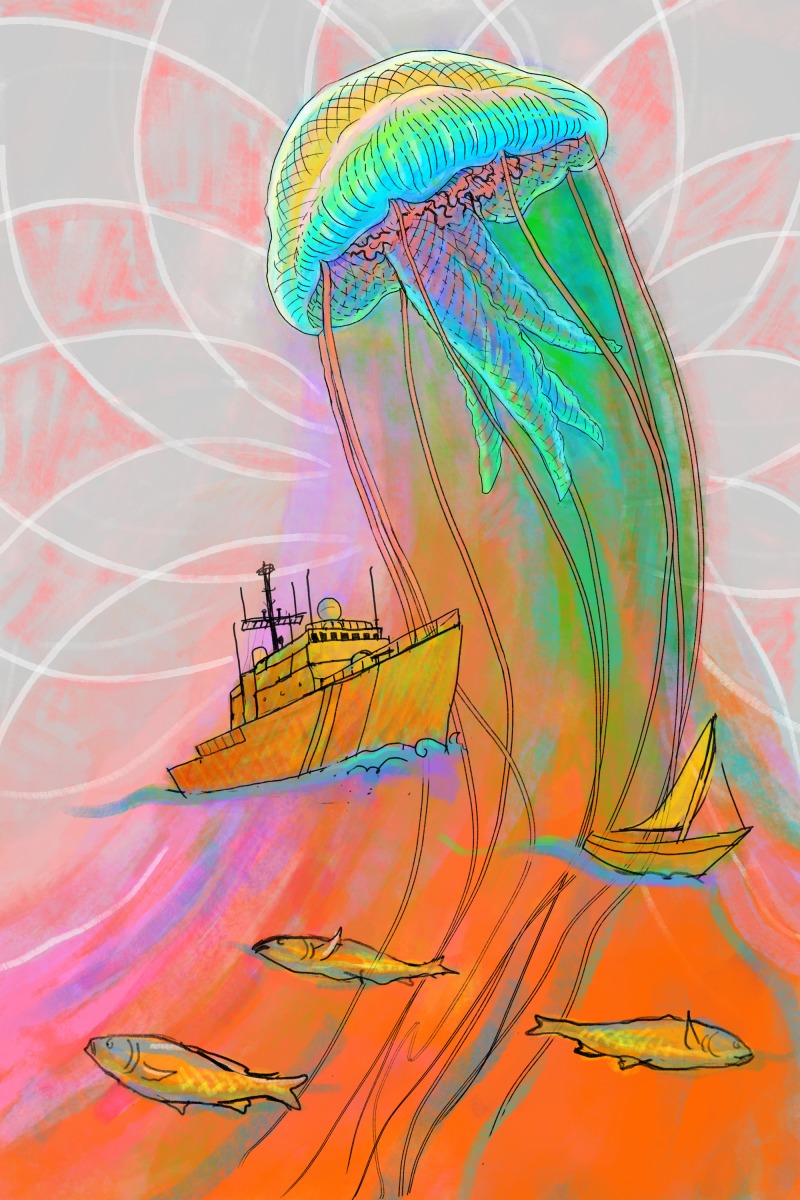
Watching jellyfish can tell us a great deal about the sea.

Illustration: Ben Marder.

For a lab scientist and an unabashed city girl, it is disconcerting to discover the extent of the influence of the yearly fluctuations in water temperature on the animals that we use for experiments. All the animals we study generate reliable stomach rhythms over a fairly large temperature range, but they become dysfunctional or 'crash' at temperatures higher than those that jellyfish have previously experienced. After particularly warm winters, the animals are less sensitive to warm temperatures, even months later in the spring and summer. We have perforce become climate scientists to a small degree, as we plot correlations between the behavior of the nervous system we study and the water temperature in the harbor. Although we now see the long-term effects of winters that remain unusually warm, we would not have suspected this were we not studying the effects of short-term temperature changes. This confirms that it is impossible to divorce laboratory science from the world in which we live if we study animals that have been wild-caught, and not bred and raised in laboratories. Some of my colleagues might thus argue it is better to use laboratory strains, but doing so would remove the richness that comes from direct connections between animals and their worlds of experience.

The presence or absence of jellyfish each early summer serves as a visible reminder that beneath the placid surface of the sea, with the ebbs and flows of its tides, there is a turbulent and rich battle for survival by creatures which are rarely seen by those who walk down to the harbor to watch the sunset or enjoy the peace of the light across the water. It is ironic and sad that the spectacular beauty of the jellyfish can be a warning that the life of the harbor is threatened. It dismays me that the natural beauty of a placid ocean or a spectacular sunset over the harbor can obscure the danger signs that our natural world is at risk.

